# An extended cost-effectiveness analysis of schizophrenia treatment in India under universal public finance

**DOI:** 10.1186/s12962-016-0058-z

**Published:** 2016-07-08

**Authors:** Neha Raykar, Aditi Nigam, Dan Chisholm

**Affiliations:** Public Health Foundation of India, Plot No 47, Sector 44, Gurgaon, Haryana 122002 India; Center for Disease Dynamics, Economics and Policy, 1400 Eye St NW, Suite 500, Washington, DC 20005 USA; Department of Mental Health and Substance Abuse, World Health Organization, Geneva, Switzerland

**Keywords:** Value of insurance, Schizophrenia, India, Cost-effectiveness analysis

## Abstract

**Background:**

Schizophrenia remains a priority condition in mental health policy and service development because of its early onset, severity and consequences for affected individuals and households.

**Aims and methods:**

This paper reports on an ‘extended’ cost-effectiveness analysis (ECEA) for schizophrenia treatment in India, which seeks to evaluate through a modeling approach not only the costs and health effects of intervention but also the consequences of a policy of universal public finance (UPF) on health and financial outcomes across income quintiles.

**Results:**

Using plausible values for input parameters, we conclude that health gains from UPF are concentrated among the poorest, whereas the non-health gains in the form of out-of-pocket private expenditures averted due to UPF are concentrated among the richest income quintiles. Value of insurance is the highest for the poorest quintile and declines with income.

**Conclusions:**

Universal public finance can play a crucial role in ameliorating the adverse economic and social consequences of schizophrenia and its treatment in resource-constrained settings where health insurance coverage is generally poor. This paper shows the potential distributional and financial risk protection effects of treating schizophrenia.

**Electronic supplementary material:**

The online version of this article (doi:10.1186/s12962-016-0058-z) contains supplementary material, which is available to authorized users.

## Background

Schizophrenia poses a considerable public health and social policy challenge because of its severity, its often catastrophic effect on the welfare and income of family members and the significant risk that patients will suffer severe human rights violations. The provision and effective coverage of care and treatment for severe mental disorders such as schizophrenia in India is limited, both in terms of access to services and financial protection or insurance. Efforts to scale up community-based public mental health services can contribute strongly to greater equality of access, since such services will serve more people in need and with less reliance on direct out-of-pocket spending.

This paper considers this claim through an innovative approach to economic evaluation called ‘extended cost-effectiveness analysis’ [1]. ECEA goes beyond conventional cost-effectiveness analysis (CEA) by not only considering the distribution of costs and outcomes across different socioeconomic groups in the population, but also by explicitly examining the extent to which interventions or policies protect households against the financial risk of medical impoverishment [1, 2]. Taking into account current debates in India about appropriate pathways towards universal health coverage, we focus in this paper on the impact of enhanced public financing and provision of schizophrenia treatment on health and financial outcomes, including increased uptake of treatment (leading to more health gain), reduced out-of-pocket treatment costs and greater insurance against catastrophic health expenses. Assessment of these various consequences provides new, evidence-based insights to national policy makers responsible for setting priorities and allocating resources in the health sector.

ECEA builds on the evidence already generated through the standard application of cost-effectiveness analysis to schizophrenia treatment in resource-constrained developing countries, in which the focus is on the relative costs per unit of health gain. Patel et al. reviewed the cost-effectiveness of select mental disorders and conclude that first-generation antipsychotic drugs in low-income and middle-income countries and their benefits can increase through psychosocial treatments at the community level [3]. Chisholm et al. analyzed the cost-effectiveness of first- and second-generation antipsychotic drugs—alone or in combination with psychosocial counseling—in Chile, Nigeria and Sri Lanka and found that community-based outpatient provision of older (first-generation) antipsychotic drugs and psychosocial treatment is the most cost-effective intervention. Moreover, the estimated cost of increasing treatment coverage through a community-based service model together with efficient treatment options is very low (investment of <Int.$1 per capita) [4]. Chisholm and Saxena evaluated the comparative costs and effects of a package of interventions in sub-Saharan Africa and Southeast Asia for a cluster of five neuropsychiatric conditions, including schizophrenia and found that neuroleptic antipsychotic drugs and psychosocial treatment were the most cost-effective intervention for schizophrenia [5]. Phanthunane et al. surveyed patients seeking treatment for schizophrenia in Thailand to provide detailed breakdown of the costs involved in schizophrenia treatment including health care costs and productivity losses to patients and families [6]. In Kenya, de Menil et al. find that a community based intervention is cost-effective and achieves increasing returns over time but may not be affordable for the Kenyan government [7]. Cost-effectiveness studies on chronic schizophrenia treatments have also compared the efficacy of typical (first-generation) versus atypical (newer) drugs in low-resource settings, such as China and Thailand [8, 9]. Clinical studies from high-income country settings evaluate cost-effectiveness and compliance rates of first- versus second-generation antipsychotic drugs using pharmaco-economic analysis and find the latter to have a higher cost-effectiveness and better safety profile [10, 11].

## Methods

### Analytical approach

We constructed an equation-based, static (single year) model of the expected costs, health outcomes and financial risk protection effects (including averted private out-of-pocket expenditures) associated with a policy of enhanced public financing of schizophrenia treatment in India. While UPF in India has previously been available for specific diseases such as AIDS, it has recently moved toward secondary and tertiary care insurance coverage in the form of programs such as Rashtriya Swasthya Bima Yojana aimed at providing financial protection against catastrophic health expenses. However, such schemes currently exclude treatment of mental disorders and consequently, there has been no quantitative assessment of the amount of financial protection that a UPF scheme can provide against schizophrenia treatment. Since this is the first application of ECEA to be carried out in the context of mental disorders, our first goal was to test its applicability and assess its internal validity. We accomplished this by constructing a series of equation-based ECEA models that employed the same epidemiological and treatment cost-outcome input data used in previous CEA studies in the context of South-East Asia [5]. Additional information output from the ECEA model—particularly the estimated value of financial risk protection arising from public financing of health care costs—could then be readily interpreted with reference to this earlier published work. We subsequently adapted or updated model input parameters to better reflect the current situation and evidence base for schizophrenia treatment in India (the model is available from the authors on request).

### Interventions modeled

In this model, all persons treated for schizophrenia in non-specialized health care settings receive a combination of first-generation antipsychotic drugs as well as basic—or, for a small proportion, intensive—psychosocial treatment. First-generation antipsychotic medication is either tablet doses of haloperidol-chlorpromazine or an injection of fluphenazine, with biperiden administered for side effects. The second-generation antipsychotic medication considered is tablets of risperidone. We use a weighted average of these anti-psychotic medications with basic psychosocial treatment as a single treatment package. After the implementation of UPF, 80 % of the population that needs treatment would receive publicly financed care (target coverage). The average treatment coverage rate for schizophrenia is 40 % based on a World Health Survey study from 2003 on coverage of schizophrenia treatment in 6 states in India [12]. Due to increased detection and healthcare utilization rates among the richer socioeconomic groups, we distribute the coverage rates to range from 30 % in the poorest income group to 50 % in the richest.

### Treatment costs

For this model, all patients treated for schizophrenia are assumed to receive a combination of antipsychotic drugs and basic psychosocial treatment; 90 % receive haloperidol or chlorpromazine with biperiden (for side effects) and the remaining 10 % receive a fluphenazine injection. Fifteen percent are assumed to receive inpatient psychiatric care in a short-term community-based psychiatric unit, 2 % are long-term community-based residential patients and 50 % have outpatient visits (for follow-ups) [13]. Finally, 10 % of treated patients are modeled to receive more intensive, individual-based psychological treatment. Table 2 presents the costs of all the treatment resources used as inputs in the model. Average per unit treatment costs are sourced from the mhGAP costing tool for India, based on the study site of Sehore district in Madhya Pradesh, India. The mhGAP costing tool values tradable healthcare goods such as medicines and diagnostic equipment at international prices adjusted for shipping and distribution costs. Default drug prices are from the International Drug Price Indicator Guide. Costs of non-tradeable healthcare goods in the form of local personnel, in-patient and out-patient care, consumables, building costs and utilities are predicted using the WHO-CHOICE costing database [13]. As Table 2 indicates, the average annual schizophrenia treatment cost per case under a policy of universal public finance is approximately $177 (2012 USD). This does not include direct non-medical costs such as transport, time and opportunity costs.

### Health impacts

We use disability-adjusted life years (DALYs) as the health outcome to evaluate the consequences of enhanced public financing for schizophrenia treatment. However, since schizophrenia treatment is accorded with no direct mortality effect, DALYs were estimated using the prevalent YLD method:

$${\text{YLD }} = {\text{prevalent cases }}*\; \left( {{\text{effect size }}*{\text{ adherence}}} \right) \, *{\text{ disability weight }}*{\text{average duration of disability }}*{\text{ target coverage}}$$

Data on schizophrenia prevalence rates are from the Global Burden of Disease study’s 2010 DisMod-MR output [14]. These rates are stratified by region, age group and gender. Disability weights for residual and acute cases are 0.576 and 0.756 respectively, where zero refers to no disability [15]; we use a weighted average composite disability weight based on time spent in a state of acute (20 %) versus residual (80 %) schizophrenia, where acute specifically refers to time spent in a continuous florid state (typically lasting 4–8 weeks). Our definition of acute schizophrenia and the associated distribution of time spent in this acute state is different to that reported by Ferrari et al. [16] who estimate on the basis of six studies that 63 % of cases (not time spent) are acute and only 37 % of cases are residual. Our composite disability weight of 0.61 is very much in line with WHO and other previous estimates of the average level of disability associated with schizophrenia. To calculate improvements in disability compared to (untreated) natural history, treatment effect sizes reported in the literature were converted into an equivalent change in disability weight [4]; this resulted in a proportionate improvement in the disability weight of 24 % [13, 17, 18]. Adherence to treatment is set to be 76 % for treatment with first- or second-generation mediation [19].

### Value of insurance

Following Verguet et al. [1] we calculate the expected value of a gamble concerning the cost of treating schizophrenia without UPF at the individual level, as follows:

$$Y_{p} = \, y\left( {1 - p} \right) \, + \, p\left( {y - c} \right)$$

where p = overall probability of receiving care for schizophrenia (calculated as, coverage*prevalence rates per quintile), c = treatment cost and y = income. Risk aversion is used here as a characteristic of people’s utility function. Specifically, to estimate financial risk protection of the policy, we first estimated the individual’s expected income before public financing, which depends on treatment coverage and associated out-of-pocket costs. An individual’s certainty equivalent is then estimated by assigning individuals a utility function that specifies their risk aversion, which is equivalent to calculating their willingness to pay for insurance against the risk of medical expenditure. The certainty equivalent, assuming a coefficient of relative risk aversion *r*, is

$$Y* = \, \left[ {\left( {1 - p} \right)y^{1 - r} + \, p\left( {y - c} \right)^{1 - r} } \right]^{1/1 - r}$$

Money metric value of insurance *v(p, y, c)* at the individual level is then

$$v\left( {y,p,c} \right) \, = \, Y_{p} {-} \, Y*$$

The total insurance value per quintile of income is

$$\Delta \left( v \right) \, * \, quintile \, size \, * \, target \, coverage$$

where target coverage is assumed to be 80 %. The value of 3 is considered to be an appropriate, conservative level of risk aversion [20, 21].

### Analysis of the distribution of costs and effects across income groups

To perform a quintile-based income analysis, we assume an evenly distributed cohort size of 200,000 individuals in a population of 1 million. Concerning prevalence, we apply DisMod’s epidemiological indicators for South Asia to a large household survey in India: Round 3 of the District Level Household and Facility Survey [22]. DLHS-3, conducted in 2007–08, is a nationally representative data set on reproductive and child health indicators, covering 720,320 households and over 3.7 million individuals from 601 districts across India [22]. The survey reports demographic information (age, gender, socioeconomic status) for each member of the household and also computes a wealth quintile for each individual based on her household’s composition of owned assets. To each such individual in the DLHS-3 sample, we assign the relevant schizophrenia prevalence rate from DisMod, based on the individual’s age and gender. Thus, the DLHS-3 sample now has information on every individual’s prevalence rate for schizophrenia, besides her gender, age in completed years and wealth quintile. Using sampling weights, we then derive a weighted average prevalence rate for each income quintile, assuming the DLHS-3 wealth quintiles are a good approximation of the income quintile distribution in India. The prevalence rates per income quintile are presented in Table 1. As seen in the table, these rates increase with higher income groups. This is a reflection of the demographic composition of the DLHS-3 sample. Data from DisMod indicates that the age-group with the highest rate of prevalence is 20–24 years followed by 25–34 years for both males and females. Demographic composition of the DLHS-3 sample suggests that proportions of these two high-prevalence age groups increase with higher income quintiles; individuals in these age-groups together constitute 20 % of the poorest quintile, 22 % of the second quintile, 24 % of the middle quintile, 26 % of the richer quintile and 28 % of the richest quintile. Thus, the observed increase in prevalence rates with income is because the relative sample shares of the age-groups with the highest prevalence rates increase with income owing to the demographic composition of the survey data.

**Table 1:** Parameters used for UPF of schizophrenia treatment and their corresponding sources

Input	Value					Source
*Demography*						
Cohort size		1,000,000				Authors’ assumption
Cohort size per quintile		200,000				Authors’ assumption
*Treatment impact*						
a. Population-wide						
Coefficient of relative risk aversion		3				[20, 21]
Disability weight (residual state)		0.576				[15]
Disability weight (acute state)		0.756				[15]
Treatment effectiveness (anti-psychotic medication + psychosocial treatment)		24 %				[5]
Treatment adherence rate		76 %				[19]
b. Quintile-specific	I	II	III	IV	V	
Current coverage	30 %	35 %	40 %	45 %	50 %	[27]
Target coverage	80 %	80 %	80 %	80 %	80 %	Authors’ assumption
Prevalence rates per quintile	0.25 %	0.26 %	0.27 %	0.29 %	0.32 %	[14, 22]
Overall probability of seeking care	0.08 %	0.09 %	0.11 %	0.13 %	0.16 %	Authors’ calculations
*Income*						
Average monthly GDP per capita income (current USD)	$641	$911	$1177	$1562	$3211	[23]

For calculating current average monthly per capita gross domestic product (GDP) per income quintile, we used the World Bank’s Poverty Calculation Net (PovCalNet) tool [23], which reports the share of each decile as a proportion of the total monthly consumption of India based on a sample of households surveyed in 2009. The consumption shares by decile are based on estimated Lorenz curves; households are ranked by consumption per person and distributions are population (household size and sampling expansion factor) weighted. Based on the consumption shares, which range from 3.7 % in the lowest decile to 28.8 % in the highest decile, we create a multiplier for each quintile based on its relative share of consumption. This is done by taking each quintile’s percentage share of consumption and dividing it by the average percentage share across all the quintiles. This gives us a relative weight for each quintile that is further multiplied by the annual GDP per capita of $1500 (current USD) to calculate the average annual GDP per capita for each quintile, which ranges from $641 in the poorest quintile to $3211 in the richest quintile (Table 1) [23].

To evaluate the consequences of UPF for private (OOP) expenses toward schizophrenia treatment, we first estimate the quintile-specific total treatment costs under current coverage rates as well as under the scenario where UPF targets 80 % coverage. We assume that at least 70 % of the total treatment costs are borne OOP by individuals across all quintiles [24, 25]. We then estimate the quintile-specific OOP expenses incurred by individuals in the absence of UPF and those averted under UPF’s target coverage of 80 %. We also report the additional costs to the government from providing UPF across quintiles.

## Results

### Cost-effectiveness

In this section, we present quintile-based outcomes of standard cost-effectiveness of UPF relative to the current treatment scenario for schizophrenia, where treatment is a combination of first- and second-generation antipsychotic medication with basic psychosocial treatment (Table 3). Regarding health gains from UPF: extending schizophrenia treatment from baseline coverage rates (ranging from 30 to 50 % across quintiles) to a target coverage rate of 80 % for all quintiles under UPF, averts an extra 22 to 28 DALYs per quintile population of 200,000 persons, for a total of 122 averted DALYs per one million population. The estimated burden of disease per one million population is 1704 DALYs and the currently averted burden is 126 DALYs, so this is equivalent to doubling the averted burden (from 7 to 14 % of disease burden).

As seen in Table 2, the total annual cost of treatment per case is USD $177.42. In the absence of UPF, the total annual costs of treatment per quintile range from $26,721 in the poorest to $57,059 in the richest quintile. Extending treatment to 80 % of the population increases the total annual treatment costs, ranging from $71,257 in the poorest quintile to $91,295 in the richest quintile. The cost per capita is $0.36 for the poorest quintile and $0.46 for the richest quintile. This yields a cost- effectiveness ratio of USD $1589 per DALY averted for each income quintile. The cost-effectiveness ratio is stable across income groups because the extra cost associated with reaching target coverage in the lower income quintiles is offset by a commensurate improvement in health outcomes (Table 2).

**Table 2:** Treatment resource costs and shares of out-of-pocket (OOP) private expenditure

Treatment resource costs	% of cases needing	Quantity per service user (per year)	Unit cost (price) ($)	Cost per case ($)
*I. Primary health center*				
Anti-psychotic medication primary care visits	100	4	1.78	7.12
Basic psychosocial treatment	100	6	1.78	10.68
Intensive psychosocial treatment	10	18	5.56	10.01
*II. Hospital*				
Outpatient visits for short term inpatients	50	12	2.51	15.06
Inpatient treatment- psychiatric unit-short term	15	28	8.83	37.09
Inpatient treatment- residential unit-long term	2	180	8.47	30.49
*III. Drug*				
Chlorpromazine	25	1095	0.01	3.67
Haloperidol	50	584	0.00	1.17
Risperidone	10	913	0.07	7.95
Fluphenazine	10	12	0.60	0.96
Biperiden	10	70	0.10	0.94
*IV. Other*				
Lab tests	50	1	5.00	2.50
*Total cost per case (2012 USD/year)*				*177.42*

As discussed earlier, in the absence of UPF, private individuals in India incur a large share of total treatment costs. With UPF, this cost burden will be transferred to the government. The impact of UPF on private costs averted is shown in Table 3. The total OOP private expenditure averted due to UPF is estimated at $276,623. Moreover, it increases across income quintiles, ranging from $49,880 for the poorest quintile and $63,906 for the richest quintile. OOP expenditures are mainly a function of prevalence rates, coverage rates and the percentage share of health expenses that are out-of-pocket and unit cost per case. Since unit cost of treatment per case ($177. 4) and the percentage of total expenses that are paid OOP (70 %) are assumed constant across all quintiles, this upward trend in OOP expenses averted from UPF is mainly a reflection of average prevalence rates rising with income and the gradient of current coverage rates across quintiles as seen in Table 1.

**Table 3:** Results

Outcome	Income quintile I	Income quintile II	Income quintile III	Income quintile IV	Income quintile V	Total
YLD (current burden)	307	316	333	354	394	1704
DALY averted by UPF (averted burden)	28	26	24	23	22	122
*Current coverage*						
Total costs of treatment ($)	26.721	32.042	38.666	46.156	57.059	200.644
Private costs of treatment ($)	18.705	22.429	27.066	32.309	39.942	140.451
Current costs met by government ($)	8.016	9.613	11.600	13.847	17.118	60.193
*Target coverage (under UPF)*						
Total costs of treatment ($)	71.257	73.238	77.331	82.055	91.295	395.176
Additional costs to government ($)	44.535	41.196	38.666	35.899	34.236	194.532
OOP expenses averted ($)	49.880	51.267	54.132	57,439	63,906	276.623
Cost-effectiveness ratio (Cost/DALY averted) ($)	1.589	1.589	1.589	1.589	1.589	
Insurance value ($)	7.282	5.587	4.972	4.302	2.439	24.582

UPF = universal public financing for 80 % of population in need. Results are based on a population of 1 million people, with intervention benefits equally divided among income quintiles of 200,000 persons each (quintile I having the lowest household income and quintile V, the highest). “Target coverage” of UPF for schizophrenia treatment for all income groups was set at 80 %. All monetary values or costs are expressed in U.S. 2012 dollars. “Total costs” = (direct government expenditures) + (private expenditures, including out-of-pocket costs). “Insurance value” = financial risk protection provided (based on current coverage)

Under this scenario, if 80 % of a population of one million is targeted for coverage through UPF, the government must meet a total cost of $395,176 per one million individuals.

**Fig. 1:**
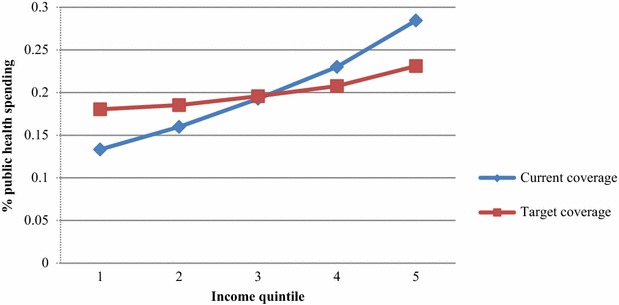
Distribution of spending on schizophrenia treatment

Figure 1 compares the quintile distribution of public health spending on schizophrenia treatment under current coverage (ranging from 30 to 50 %) versus that under UPF’s target coverage of 80 %. Under current coverage, distribution of public health spending accords with evidence in Mahal et al. of a regressive pattern of health spending that disproportionately favors the rich; this is mainly a manifestation of higher treatment coverage rates among the richer groups [24]. Introduction of UPF flattens this distribution, thus creating distributional consequences of universalizing healthcare coverage and costs.

### Value of insurance

**Fig. 2:**
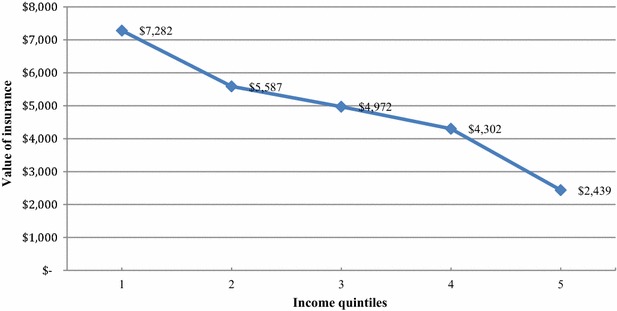
Money metric value of insurance under UPF, by income quintile

An additional financial consequence of a health intervention such as UPF is the money metric value of insurance, which is the amount an individual is willing to pay to receive risk protection i.e., to be in a healthy state vis-à-vis a poor health state. The total annual value of insurance from UPF for the entire population of 1 million is USD $24,582. The value is the highest for the lowest income quintile and decreases as income rises. The annual insurance value for the poorest quintile is USD $7282, which is approximately 30 % of the total value (Fig. 2). The second income quintile has an annual insurance value of USD $5587; the third income quintile, $4972; and the fourth income quintile, $4302. The highest income quintile has an annual insurance value of USD $2439, which is approximately 10 % of the total value.

### Uncertainty analysis

To test the reliability of our model predictions based on plausible ranges of the input parameters derived from various sources, we performed an uncertainty analysis with 1000 simulations [26]. Under the Latin hypercube sampling (LHS) scheme, a probability distribution is constructed for each input parameter that is not known with certainty. Input parameters that are known with large certainty, either because they are fixed values constructed for the simplicity of the model (such as population/cohort size) or because they are reliable values derived from large, nationally representative Indian survey datasets are excluded from the model. Table 4 describes the baseline values and the assumed range and distributions for those input parameters that are likely to be affected by uncertainty, either because they are aggregate, regional estimates not specific to India, or because they are based on local village-level field studies with small sample sizes that may not be representative of the country as a whole. In case of current coverage rates, we consider two alternative average current coverage rates of 20 % (pessimistic case) and 50 % (optimistic case) in Table 4. We specified a plausible range of minimum and maximum values for the uncertain parameters based on available literature or on assumptions made by the working team. Figure A1 shows the summary statistics from the LHS scheme with 1000 simulations for three outcomes—DALY averted, OOP expenses averted and money metric value of insurance—in the form of box plots representing quartile values for each income quintile. Please see Additional file 1 for the box plots. We do not show the corresponding boxplots for cost-effectiveness ratio here since the ratio is constant across quintiles. The summary statistics for this outcome are included in Table 3. Tables 5, 6 present the values including means for all four outcomes in tabular form. Reducing current coverage by half, for example, reduces the current averted burden (as well as the value of insurance) by 50 %, but also reduces the costs and averted private expenditure associated with current treatment by this proportion, thereby resulting in no change in cost-effectiveness. It is worth mentioning that the relatively larger variation across quintiles for value of insurance is most likely due to the variation in prevalence rates, which range from 0.251 % (Q1) to 0.322 % (Q5). This variation in prevalence is due to the age distribution of the DLHS-3 sample as explained earlier.

**Table 4:** Input parameters with uncertainty

Input parameters	Probability distribution	Baseline values	Max	Min	Source
Disability weight (residual)	Uniform	0.58	0.76	0.40	[15]
Disability weight (acute)	Uniform	0.76	0.89	0.57	[15]
Treatment efficacy (anti-psychotic + psychosocial treatment)	Uniform	0.24	0.26	0.22	[13, 17, 18]
Treatment adherence rate	Uniform	0.76	0.84	0.68	[19]
Total cost per case ($)	Uniform	177.42	212.90	141.93	[13]
*Current coverage rate by income quintile*—*pessimistic case*					
I	Uniform	0.10	0.20	0.00	
II	Uniform	0.15	0.25	0.05	
III	Uniform	0.20	0.30	0.10	
IV	Uniform	0.25	0.35	0.15	
V	Uniform	0.30	0.40	0.20	
*Current coverage rate by income quintile*—*optimistic case*					
I	Uniform	0.40	0.50	0.30	
II	Uniform	0.45	0.55	0.35	
III	Uniform	0.50	0.60	0.40	
IV	Uniform	0.55	0.65	0.45	
V	Uniform	0.60	0.70	0.50	
*Target coverage rate by income quintile*					
Quintiles I–V	Uniform	0.80	0.90	0.70	Author’s assumption
*Percentage of all costs that are Out Of Pocket (OOP) by income quintile*					
Quintiles I–V	Uniform	0.70	0.80	0.60	Author’s assumption

**Table 5:** Summary statistics of Latin hypercube distribution for ECEA outcomes—pessimistic case

Outcome	DALY averted					
Income quintile	I	II	III	IV	V	Total
Minimum	20.97	19.37	19.23	17.31	17.56	111.52
1st quartile	33.18	31.46	30.64	29.70	30.01	160.00
Mean	39.02	37.27	36.29	35.26	35.66	183.50
Median	38.78	36.80	35.51	34.53	35.24	181.68
3rd quartile	44.02	42.56	41.31	40.04	40.78	205.15
Maximum	74.05	66.25	64.94	59.57	62.07	295.10

Outcome	OOP expenses averted					
Income quintile	I	II	III	IV	V	Total
Minimum ($)	31.861	31.607	33.301	36.283	41.366	198.745
1st quartile ($)	43.972	45.189	47.408	50.907	56.564	248.738
Mean ($)	49.884	51.277	54.128	57.447	63.880	276.617
Median ($)	49.281	50.822	53.314	56.710	63.165	275.072
3rd quartile ($)	55.488	56.774	60.124	63.478	70.511	304.159
Maximum ($)	73.910	77.080	80.511	84.425	94.717	369.918

Outcome	Cost-effectiveness ratio (USD/DALY averted)					
Income quintile	I	II	III	IV	V	Total
Minimum ($)	940	940	940	940	940	940
1st quartile ($)	1.399	1.399	1.399	1.399	1.399	1.399
Mean ($)	1.641	1.641	1.641	1.641	1.641	1.641
Median ($)	1.605	1.605	1.605	1.605	1.605	1.605
3rd quartile ($)	1.861	1.861	1.861	1.861	1.861	1.861
Maximum ($)	2.847	2.847	2.847	2.847	2.847	2.847

Outcome	Value of insurance					
Income quintile	I	II	III	IV	V	Total
Minimum ($)	1	331	551	618	409	3.335
1st Quartile ($)	901	1.189	1.354	1.354	880	6.825
Mean ($)	2.015	1.974	2.046	1.965	1.195	9.194
Median ($)	1.772	1.757	1.850	1.839	1.144	8.819
3rd Quartile ($)	2.848	2.600	2.580	2.411	1.446	11.111
Maximum ($)	7.945	6.717	5.902	5.211	2.969	22.213

**Table 6:** Summary statistics of Latin hypercube distribution for ECEA outcomes—optimistic case

Outcome	DALY averted					
Income quintile	I	II	III	IV	V	Total
Minimum	9.81	6.90	6.18	2.90	0.97	45.85
1st Quartile	17.94	15.63	13.74	11.75	9.89	77.06
Mean	22.27	20.06	18.16	16.01	14.22	90.71
Median	21.97	19.52	17.83	15.98	13.63	89.35
3rd Quartile	26.06	23.88	21.89	19.82	18.30	102.60
Maximum	41.74	40.48	43.57	35.49	35.41	184.75

Outcome	OOP expenses averted					
Income quintile	I	II	III	IV	V	Total
Minimum ($)	31.630	32.451	35.402	37.265	41.665	197.982
1st Quartile ($)	43.554	45.019	47.893	50.752	56.518	247.803
Mean ($)	49.904	51.307	54.091	57.459	63.898	276.659
Median ($)	49.491	51.217	53.688	57.160	63.382	276.757
3rd Quartile ($)	55.316	57.303	59.998	63.395	71.026	303.456
Maximum ($)	74.272	74.800	81.295	86.530	97.244	380.129

Outcome	Cost-effectiveness ratio (USD/DALY averted)					
Income quintile	I	II	III	IV	V	Total
Minimum ($)	938	938	938	938	938	938
1st Quartile ($)	1.401	1.401	1.401	1.401	1.401	1.401
Mean ($)	1.640	1.640	1.640	1.640	1.640	1.640
Median ($)	1.613	1.613	1.613	1.613	1.613	1.613
3rd Quartile ($)	1.848	1.848	1.848	1.848	1.848	1.848
Maximum ($)	2.728	2.728	2.728	2.728	2.728	2.728

Outcome	Value of insurance					
Income quintile	I	II	III	IV	V	Total
Minimum ($)	2.762	2.038	1.918	1.671	912	12.585
1st Quartile ($)	5.723	4.315	3.808	3.186	1.802	19.783
Mean ($)	8.069	5.918	5.096	4.314	2.393	25.790
Median ($)	7.589	5.638	4.805	4.112	2.306	25.166
3rd Quartile ($)	9.964	7.193	6.097	5.251	2.883	31.456
Maximum ($)	19.465	13.911	11.010	10.122	5.084	46.800

## Discussion

While existing research considers the cost-effectiveness of schizophrenia treatment in low-resource settings including India, our paper considers the extension of the current literature to evaluate the cost-effectiveness of publicly financed schizophrenia treatment in India and includes the non-health financial benefits of UPF to private individuals or households. Our results show that when current coverage is extended to 80 % under UPF, health gains in terms of DALYs averted from UPF are the highest among the poor; however, these gains come at a higher cost for the poorest quintile, since UPF covers a larger proportion of this income group vis-à-vis the richest.

The money metric value of insurance is a quantifiable measure of financial protection under UPF and this ECEA illustrates that it is feasible to design essential packages of publicly financed health services to include financial protection as an additional outcome besides health gains. We see a relatively modest downward trend in the insurance values from the lowest income quintile to the highest income quintile; risk protection from UPF would accrue primarily to low-income groups. Total value of insurance is USD $24,582 to one million population, of which, 30 % accrues to the poorest income quintile.

Finally, we offer a few caveats on the results. The model remains limited in scope because of the paucity of reliable data for India on mental disorders and schizophrenia in particular. Many of the epidemiological and efficacy parameter values used in this analysis rely on regional (South Asia) estimates. Data on treatment costs come largely from the mhGAP costing tool, which is based on a small sample of individuals from a relatively small study site in India (Sehore, Madhya Pradesh); estimates on costs of services may therefore not be representative at the national level. Moreover, we did not estimate all potential costs incurred by service users, such as the transportation, time and opportunity costs involved in receiving treatment.

There are no reliable data on key input parameters disaggregated by income levels. For example, we expect treatment costs to vary by income but we do not have data on income-specific treatment costs. Likewise, we also expect treatment effectiveness and adherence rates to vary by income quintiles. Similar to treatment coverage rates, adherence rate for poorer quintiles may be lower as low-income households may face additional constraints to access care and supply issues may affect availability of drugs at the health facility level; however, lack of data on variation of treatment adherence rates by income for mental disorders in India limits our analysis to assuming a constant adherence rate in the model.

A further concern is the use of DALYs as a measure of health outcome for schizophrenia treatment. Although DALYs are a useful measure of the efficiency of schizophrenia care relative to other health investments, they do not deal with comorbidity and cannot reflect the effect of treatment on the patients’ families [17]. In this analysis, we only assessed the impact of treatment on reducing the non-fatal burden of diseases associated with schizophrenia (measured in terms of years lived with disability or YLD); although schizophrenia is clearly associated with excess mortality, effects on this dimension of disease burden (measured in terms of years of life lost) were not included since there is insufficient evidence to suggest that the psychosocial and pharmacological interventions modelled here lead directly to a reduction in mortality.

Lastly, although the paper extends standard cost-effectiveness analysis to include certain financial outcomes, we do not account for non-health benefits of treatment in the form of workforce and household productivity gains. A cost-benefit analysis would be needed to measure those effects.

## Additional file

10.1186/s12962-016-0058-z Summary statistics from LHS scheme with 1000 simulations.

## Abbreviations

DALY: disability adjusted life year
DLHS-3: district level household survey-3
ECEA: extended cost effectiveness analysis
GDP: gross domestic product
mhGAP: Mental Health Gap Action Programme
OOP: out-of-pocket
UPF: Universal Public Finance
YLD: years lived with disability
YLL: years of life lost
