# Supplementary material for: An extended cost-effectiveness analysis of schizophrenia treatment in India under universal public finance
Source: Cost Eff Resour Alloc. 2016 Jul 8;14:9. doi: 10.1186/s12962-016-0058-z (PMC4938947; doi:10.1186/s12962-016-0058-z)
Supplement: Supplementary file 1 — 10.1186/s12962-016-0058-z Summary statistics from LHS scheme with 1000 simulations. [file 12962_2016_58_MOESM1_ESM.docx]

**Figure S1. Summary statistics from LHS scheme with 1000 simulations**
